# Rhabdomyolysis secondary to hypervirulent *Klebsiella pneumoniae* infection: A case report

**DOI:** 10.1002/ccr3.6764

**Published:** 2022-12-21

**Authors:** Naoko Niimi, Keiko Taga, Taiju Miyagami, Toshio Naito, Chieko Mitaka

**Affiliations:** ^1^ Department of Anesthesiology and Pain Medicine Juntendo University Faculty of Medicine Tokyo Japan; ^2^ Department of General Medicine Juntendo University Faculty of Medicine Tokyo Japan

**Keywords:** hypervirulent *Klebsiella pneumonia*, liver abscess, rhabdomyolysis, septic shock

## Abstract

Hypervirulent *Klebsiella pneumoniae* (hvKP) is recognized as a lifethreatening community‐acquired infection associated with pyogenic liver abscess. However, rhabdomyolysis secondary to hvKP infection is rare. To the best of our knowledge, we report the first case of rhabdomyolysis due to hvKP infection in a patient who survived septic shock syndrome.

## Abstract 2

Longer duration of PMX‐DHP improves hemodynamics and decreases vasopressors rapidly compared to the conventional duration. Additionally, it encourages clinicians to suspect other causes of rhabdomyolysis.

## 1 BACKGROUND

*Klebsiella pneumoniae*, a gram‐negative bacillus, has been implicated as a cause of pneumonia, urinary tract infection, abdominal cavity infection, and intravascular device infection; this type of *Klebsiella pneumoniae* is recognized as classical *Klebsiella pneumoniae* (cKP).^1^ However, different types of *Klebsiella pneumoniae* have been recognized, starting in East Asia. The first report of a unique *Klebsiella pneumoniae* was reported in Taiwan in the 1980s, with characteristic of community‐acquired infection with complications of pyogenic liver abscess and endophthalmitis.^2^ This new type of *Klebsiella pneumoniae* has been named hypervirulent *Klebsiella pneumoniae* (hvKP) based on its malignancy. The features of hvKP have been described as community‐acquired infection among healthy young individuals, which are known to have unique complications of septicemia, pyogenic liver abscess, endophthalmitis, multiple abscesses, and metastatic spread of abscesses to distant sites.^1^ It has been reported that 90.9% of the pathogens causing liver abscesses with *Klebsiella pneumoniae* infection are hvKP.^3^ The string test, which uses an inoculation loop to generate a viscous string from a bacterial colony, is used for differential diagnosis between cKP and hvKP. If a string length of >5 mm is generated because of hypermucosviscosity, the test is considered positive.^4^ However, the specificity of the string test and hvKP varies between 51% and 98%, and even cKP shows positive string test rates of 17% to 23%.^5^ Although one retrospective study reported no clinical relevance of a 30‐day mortality rate between cKP and hvKP,^6^ a review article demonstrated that hvKP is associated with a significant mortality rate of 3% to 55% compared to cKP.^1^ Iron acquisition has been described as the decisive factor of hvKP's hypervirulence compared to cKP.^1^

Although various complications have been identified in hvKP, rhabdomyolysis due to hvKP infection is rare. Indeed, to the best of our knowledge, there are two case reports published in English, describing cases of *Klebsiella pneumoniae* infection complicated with rhabdomyolysis.^7^, ^8^ However, the reported cases were not specified as hvKP. Accordingly, this article might be the first report describing rhabdomyolysis due to hvKP infection.

We present our case of rhabdomyolysis secondary to hvKP infection involving septic shock from which the patient recovered.

## 2 CASE PRESENTATION

**FIGURE 1:**
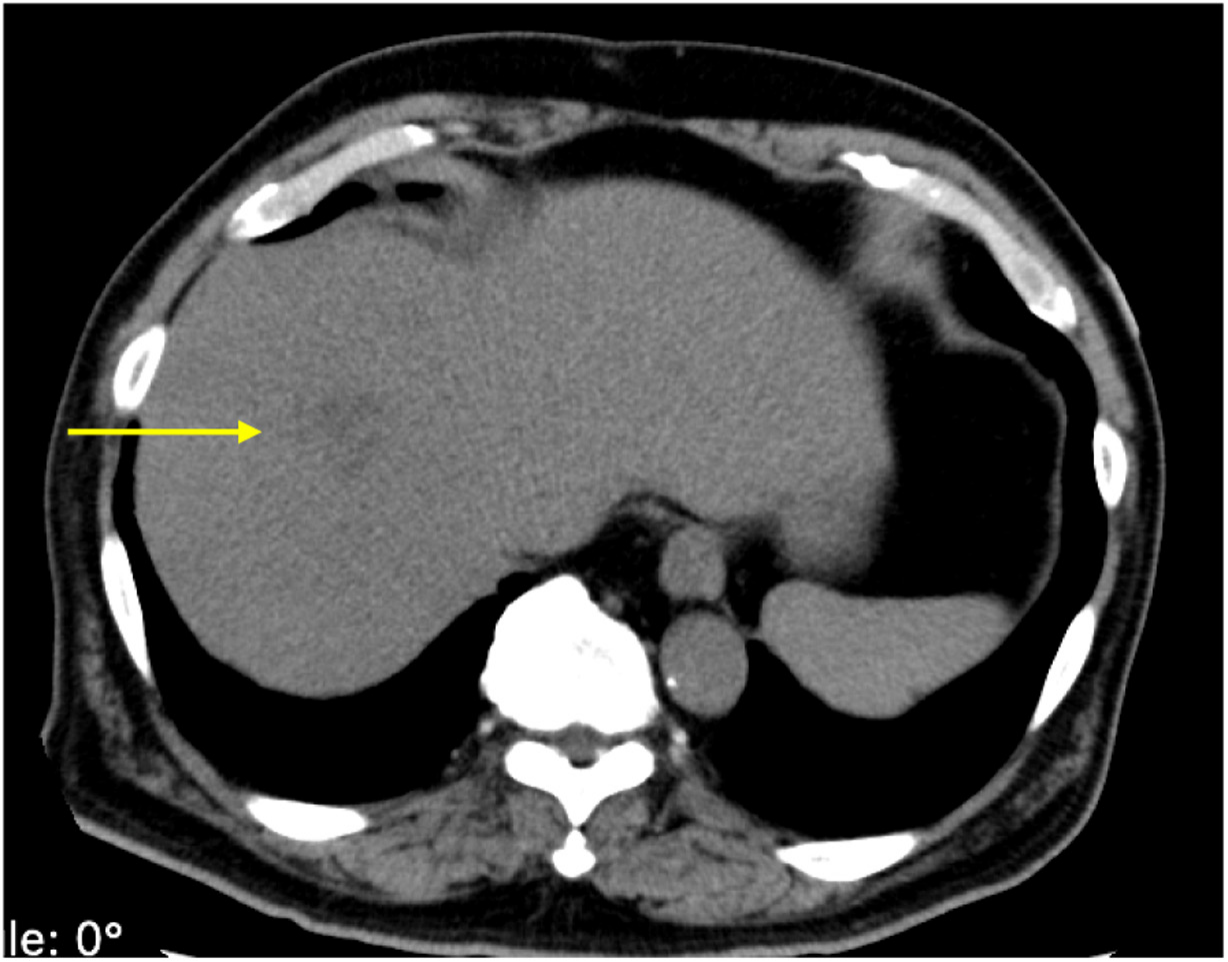
Abdominal CT scan without contrast‐enhancement on admission reveals a 26‐mm low‐density lesion.

A 68‐year‐old Japanese man (height 171 cm and weight 78 kg) with no background of immunosuppression (no history of diabetes mellitus; Hemoglobin A1c 6.1%) presented to the Outpatient Department of Juntendo University Hospital (1051‐bed university‐affiliated hospital), in Tokyo, Japan, complaining of weakness in the lower limbs, slurred speech, and lower back pain. The patient had a medical history of chronic atrial fibrillation and hypertension, for which had been treated with rivaroxaban and calcium channel blockers. On examination, he was conscious, his body temperature was 36.9°C, blood pressure was 74/59 mmHg, heart rate was 116 beats/min, and respiratory rate was 17/min. Oxygen saturation was 97% on room air. Diffuse muscle tenderness was noted in all four extremities (manual muscle test was 4/5), with numbness in the right upper and lower limbs. Initial laboratory findings revealed a leukocyte count of 10.4 × 10^9^/L (normal range of 3.9 × 10^9^–9.7 × 10^9^/L), hemoglobin of 16.5 g/dl, platelet count of 58 × 10^9^/L, creatinine of 2.88 mg/dl, and C‐reactive protein of 27.97 mg/dl. The creatinine kinase (CK) level was significantly elevated to 37,370 U/L, and the blood myoglobin level was 11,850 ng/ml (Table 1). His urine was reddish‐brown in color, but culture test was negative. Considering his renal function (estimated glomerular filtration rate of 18.2) and risk of contrast material‐induced nephrotoxicity,^9^ a non‐contrast computed tomography (CT) scan was conducted. An abdominal CT revealed a 26‐mm‐diameter hypodense lesion in the right lobe of the liver (Figure 1). Chest X‐ray, chest CT, and cranial magnetic resonance imaging showed no abnormality. Based on all the findings, the patient was diagnosed with rhabdomyolysis and was immediately started on hydration at 3000 ml/day. He was admitted to the general ward. His serum CK level decreased to 24,637 U/L and his creatinine level to 1.9 mg/dl at 9 h after admission (Table 1).

**TABLE 1:** Transitions of blood tests

	Leukocyte (3.9–9.7 × 10^9^/L)	Hb (13.4–17.1 × 10^12^/L)	Plt (153–346 × 10^9^/L)	CK (57–240 U/L)	Cre (0.6–1.0 mg/dl)	eGFR	Myoglobin (0.0–154.9 ng/ml)	CRP (0.0–0.29 mg/dl)
Admission	12.7	16.5	58	37,370	2.88	18.2	11,850	27.97
9 h after admission				24,637	1.90	28.6		
Day 4^th^	12.7	12.6	24	1345	2.23	24.0		34.63
Day 22^nd^	12.8	8.4	219	158	0.52	118.2		4.81

Abbreviations: CK; Creatinine kinase, Cre; Creatinine, eGFR; Estimated glomerular filtration rate, CRP; C‐reactive protein, Hb; Hemoglobin, Plt; platelet.

**FIGURE 2:**
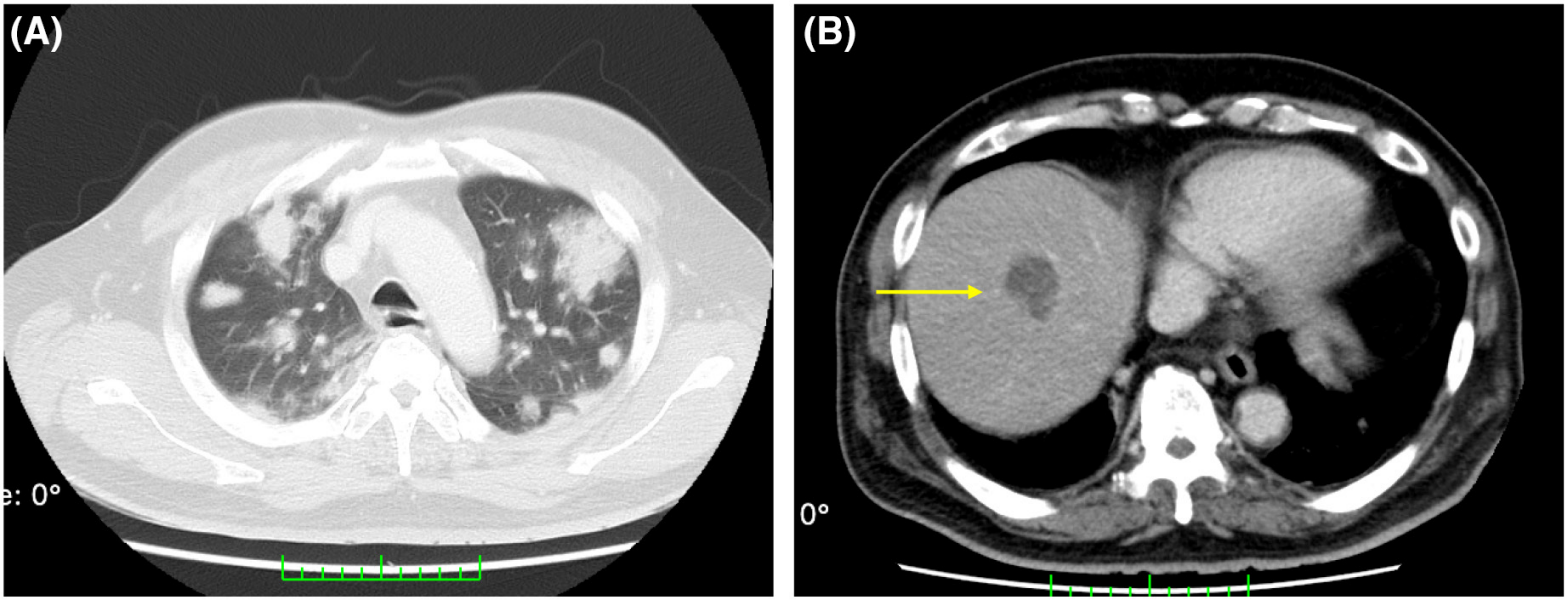
CT scan without contrast‐enhancement on the 3rd hospital day showing cotton‐like shadow and rounded infiltrate with air bronchogram; the margin of the infiltrate is fluffy, and the inside is granular in the lung (A); the liver abscess is enlarged to 33 mm on CT scan with contrast‐enhancement (B).

**FIGURE 3:**
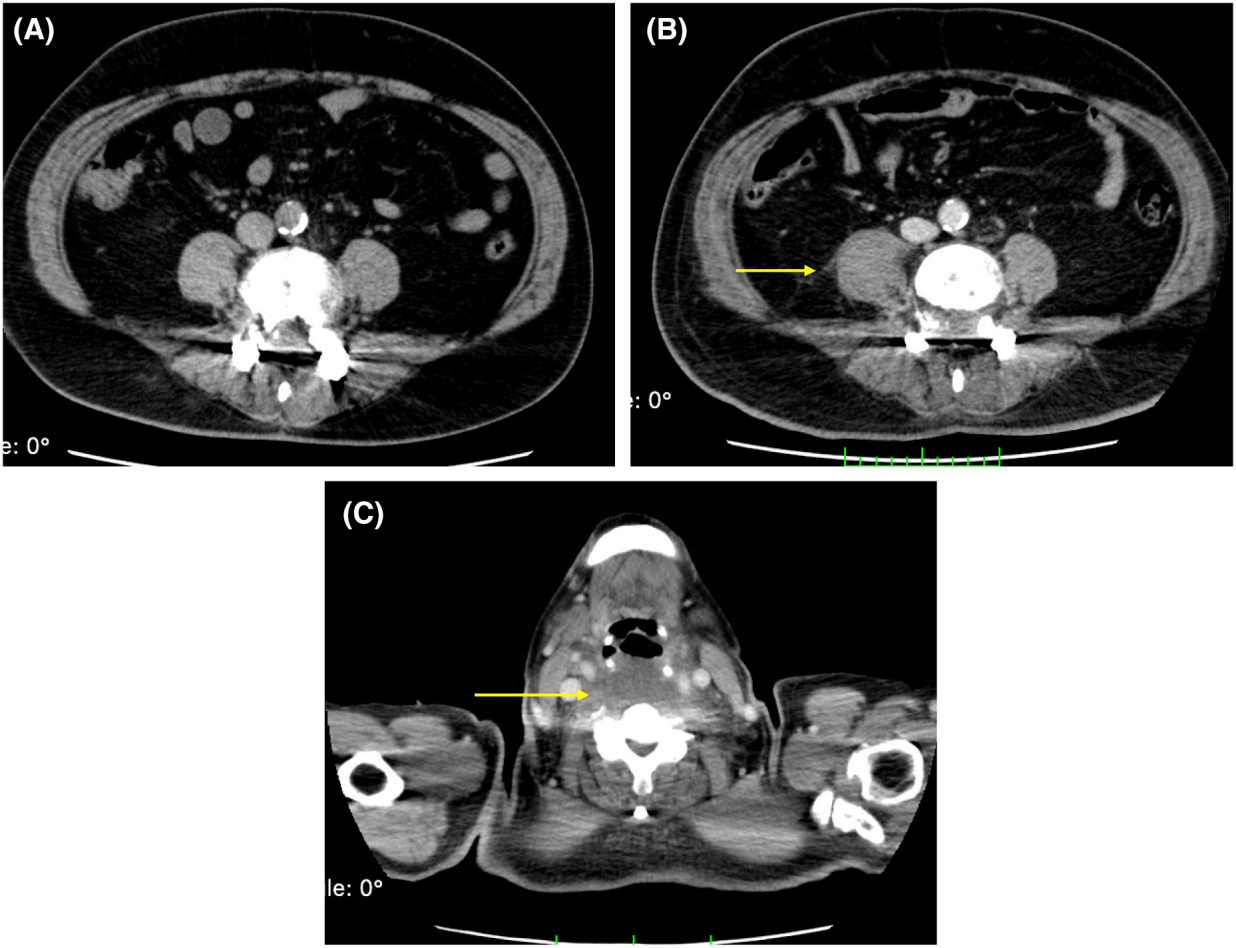
CT scan without contrast‐enhancement on the admission (A), CT scan with contrast‐enhancement on the 3rd hospital day showing an enlarged psoas major abscess (B) and retropharyngeal abscess (C).

On the 3rd hospital day, the patient developed respiratory insufficiency and was transferred to our intensive care unit. Because of hypoxemia, he was intubated, and mechanical ventilation was started. Arterial blood gas showed metabolic acidosis and hypoxemia (pH 7.30, pCO_2_ 45 mmHg, pO_2_ 84 mmHg, HCO3^−^ 22.4 mmol/L, lactate 3.2 mmol/L, under FiO_2_ 0.8). The ventilator setting of Hamilton G5 ventilator was adaptive support ventilation mode, FiO_2_ 0.8, PEEP 10 cm H_2_O, and %Min Vol 130%. Another CT scan revealed multiple newly emerged inflammatory infiltration shadows in the lung and enlarging hypodense lesions in the liver compared to that on admission (Figure 2). Newly emerged retropharyngeal abscesses and psoas major abscesses were also detected (Figure 3). Infectious endocarditis was unlikely from echocardiography which revealed mild regurgitation of mitral valve and tricuspid valve without vegetation. Therefore, two sets of blood cultures and urine cultures were performed, and intravenous meropenem (6 g/day) and vancomycin (2 g/day) and platelet transfusion were started immediately. On the 4th day, laboratory findings revealed a leukocyte count of 12.7 × 10^9^/L, platelet count of 24 × 10^9^/L, C‐reactive protein of 34.63 mg/dl, and procalcitonin of 29 ng/ml (Table 1). His body temperature increased to 40.1°C. Based on his rapidly deteriorating general status and hypotension (blood pressure: 88/50 mmHg) requiring crystalloid infusion and vasopressors (norepinephrine 0.1 μ/kg/min and vasopressin 0.03 U/min), he was diagnosed with septic shock. Both blood and urine cultures showed hvKP with a positive string test (Table 2). To adsorb endotoxin, polymyxin B‐immobilized fiber column direct hemoperfusion (PMX‐DHP) treatment was performed for twice (18 and 18.5 h, respectively). Due to such intensive care, he recovered from septic shock, vasopressors were discontinued, and pulmonary oxygenation (PaO_2_/FiO_2_) improved to 234 mmHg after PMX‐DHP treatment. Vancomycin was stopped on the 9th hospital day. Although the oxygenation of the patient has improved, mechanical ventilation was required due to severe retropharyngeal edema.

**TABLE 2:** Antibiogram of blood and urine culture

Drugs	Resistance
ABPC	R
ABPC/SBT	S
AMPC/CVA	S
PIPC/TAZ	S
CEZ	S
CTM	S
CMZ	S
CTX	S
CAZ	S
CFPM	S
IPM	S
MEPM	S
AZT	S
GM	S
AMK	S
MINO	S
CPFX	S
LVFX	S
FOM	S
ST	S

Abbreviations: ABPC; Ampicillin, ABPC/SBT; Ampicillin/Sulbactam, AMPC/CVA; Amoxicillin/Clavulanate, AZT; Aztreonam, AMK; Amikacin, CEZ; Cefazolin, CTM; Cefotiam, CMZ; Cefmetazole, CTXCefotaxime, CAZ; Ceftazidime, CFPM; Cefepime, CPFX; Ciprofloxacin, FOM; Fosfomycin, GM; Gentamicin, IPM; Imipenem, LVFX; Levofloxacin, MEPM; Meropneme, MINO; Minocycline, PIPC/TAZ; Piperacillin/Tazobactam, R = Resistant, S = Susceptible, ST; Sulfamethoxazole‐Trimethoprim.

**FIGURE 4:**
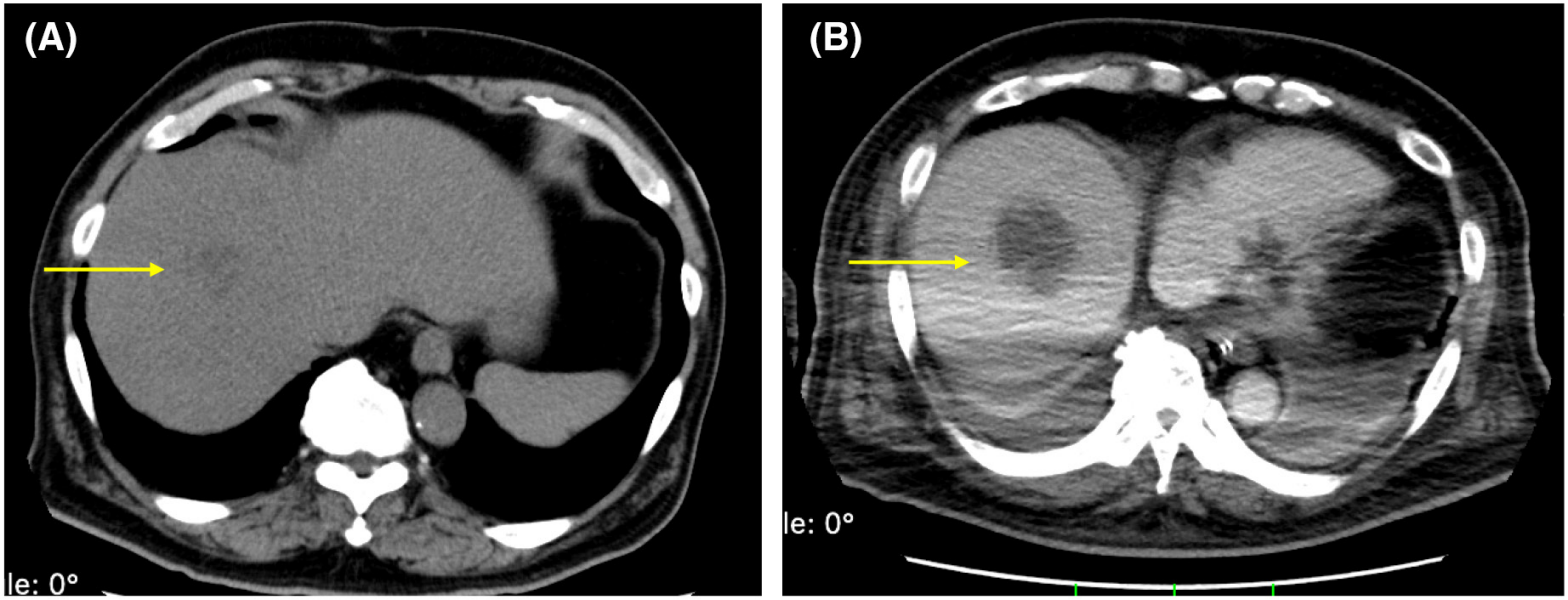
Comparison of abdominal CT scans without contrast‐enhancement on admission (A) and abdominal CT scans with contrast‐enhancement the 10th hospital day (B). The liver abscess grew from 26 (A) to 47 mm (B).

Although his general status improved, inflammation due to the multiple abscesses was prolonged. As the liver abscess was too small and the risk with percutaneous drainage at first outweighed the benefit, the abscess gradually became enlarged (Figure 4). On the 12th hospital day, ultrasonic‐guided percutaneous drainage of the liver abscess was carried out. Twenty‐five milliliters of pus was drained; the catheter placed was indwelling. Stab culture of the liver abscesses also revealed hvKP. On the 18th hospital day, drainage of the retropharyngeal abscess and tracheostomy were conducted, and he was weaned from mechanical ventilation. Finally, his CK level dropped to normal on the 22nd hospital day (Table 1). Because inflammatory mediators remained high, percutaneous drainage of the psoas major abscess was performed guided by CT scan on the 28th hospital day. After this drainage, his condition gradually improved. Meropenem was administered for almost 2 months until the extensive spread of infection disappeared.^10^ He was discharged from the intensive care unit to the general ward on the 33rd hospital day. His physiotherapy rehabilitation continued for 3 months, and he was transferred to a rehabilitation hospital on the 142nd hospital day.

## 3 DISCUSSION

Our patient had hvKP infection with a positive string test, and this infection induced multiple abscesses, such as liver abscess, retropharyngeal abscess, and psoas major abscess. He had septic shock because of severe hvKP infection. Therefore, endotoxin adsorption therapy with a longer PMX‐DHP duration was performed 2 times to treat his septic shock. PMX‐DHP has been shown to adsorb not only endotoxin but also monocytes and anandamide, resulting in a reduction in blood levels of inflammatory cytokines such as interleukin‐6, tumor necrosis factor‐alpha, and interleukin‐17A.^11^ In addition, we have shown that a longer duration of PMX‐DHP improves hemodynamics and decreases vasopressors rapidly compared to the conventional duration, and a ≥8‐h duration of PMX‐DHP was associated with an improvement in pulmonary oxygenation.^12^, ^13^ As a result, our patient's hemodynamics and pulmonary oxygenation improved after PMX‐DHP treatment, and he recovered from septic shock. It is important for clinicians to be aware of hvKP infection when treating the patient under septic shock with multiple abscesses. Whole‐body examination, string test, and all sorts of cultivation tests must be conducted when suspected.

At first, our patient presented complained of weakness in the lower limbs, slurred speech, and lower back pain. He was diagnosed with rhabdomyolysis because of a high serum CK level (37,370 U/L) and high myoglobin level (11,850 ng/ml). Rhabdomyolysis is a fatal clinical condition caused by the release of toxic intracellular content from damaged skeletal muscles. Although there are no diagnostic criteria for rhabdomyolysis, a serum CK level higher than 10 times the normal value in the absence of heart or brain diseases is used for clinical indication.^14^ Despite multiple of rhabdomyolysis, rhabdomyolysis secondary to infection is uncommon. Gabow et al.^15^ report the incidence of infectious rhabdomyolysis as 5% in their review. Furthermore, infectious rhabdomyolysis is reported to have a poorer prognosis (such as incidence of acute kidney failure) than rhabdomyolysis secondary to exercise events, though peak CK and myoglobin levels are lower in the former.^16^ The most commonly reported organisms causing infectious rhabdomyolysis are *Legionella* spp., *Streptococcus* spp., *Salmonella* spp., and influenza virus, and the main site of primary infection is the respiratory tract.^7^, ^17^ There are several hypotheses regarding the mechanism of infectious rhabdomyolysis, but none of them are well established: direct muscle invasion of bacteria or viruses, immunologic reactions such as “cytokine storms,” toxic effects from bacteria or viruses, and muscle damage caused by high fever and hypoxia.^17^ Significant mortality of 38% in infectious rhabdomyolysis has been reported.^18^

Because our patient did not have any initial imaging findings except for the hypodense lesion in the liver, we suspect that the primary source of infection was a liver abscess. To the best of our knowledge, there are only two English reports describing cases of rhabdomyolysis secondary to liver abscess,^3^, ^19^ but there are no reported rhabdomyolysis cases due to hvKP infection. Our case was rhabdomyolysis secondary to hvKP infection, making liver abscess the primary focus. Overall, this was an extremely rare case with a good course despite the high mortality rate. We believe that this patient could be saved due to multimodal therapy including antimicrobial therapy, vasopressor therapy, and mainly, longer duration of PMD‐DHP.

## 4 CONCLUSION

Both rhabdomyolysis and hvKP infection are fatal diseases. Rhabdomyolysis can be provoked by infectious diseases. This is the first case of infectious rhabdomyolysis due to hvKP infection causing septic shock. Because of the rarity of a correlation between rhabdomyolysis and infection, our case delayed in diagnosis and primary care with antibiotics. However, the patient recovered from a deteriorated condition due to our intensive care. It is important for clinicians to detect the underlying cause of rhabdomyolysis in the early phase, and if the cause of rhabdomyolysis was with hvKP infection, clinicians should be aware that the patients' condition might worsen rapidly and prepare for multimodal therapy.

## AUTHOR CONTRIBUTIONS

NN was a major contributor to manuscript writing. TM, KT, TN, and CM were responsible for care of the patient and data collection. CM edited the manuscript. All authors read and approved the final manuscript.

## FUNDING INFORMATION

None.

## CONFLICT OF INTEREST

The authors declare that they have no competing interests.

## CONSENT

Written informed consent was obtained from the patient for publication of this case report and any accompanying images. A copy of the written consent is available for review by the editor in chief of this journal.

## ETHICS STATEMENT

Not applicable.

## Data Availability

The datasets used and/or analyses during the current study are available from the corresponding author on reasonable request.
